# Drug Repurposing for COVID-19: Ethical Considerations and Roadmaps

**DOI:** 10.1017/S0963180120000481

**Published:** 2020-06-05

**Authors:** HIROYASU INO, EISUKE NAKAZAWA, AKIRA AKABAYASHI

**Keywords:** COVID-19, drug repurposing, drug repositioning, resource distribution

## Abstract

While the world rushed to develop treatments for COVID-19, some turned hopefully to drug repurposing (drug repositioning). However, little study has addressed issues of drug repurposing in emergency situations from a broader perspective, taking into account the social and ethical ramifications. When drug repurposing is employed in emergency situations, the fairness of resource distribution becomes an issue that requires careful ethical consideration.This paper examines the drug repurposing in emergency situations focusing on the fairness using Japanese cases. Ethical issues under these circumstances addressed by the authors include: maintaining the evidence level, integrity of clinical research ethics, and voluntary consent by original indication patients. In order to address these issues, they argue that rapid accumulation of ethically and scientifically valid evidence is required, as is obtaining information on resource quantity.

## Introduction

In the beginning of 2020, the World Health Organization (WHO) declared a pandemic state^1^ for Coronavirus disease 2019 (COVID-19). Although the world rushed to develop treatments and vaccines to treat the disease, some turned hopefully to drug repurposing (DR), a process of searching for existing pharmaceutical products approved for treatment of a given disease with the intent to use it as treatment or prevention of a different disease. In this process, the original disease is referred to as the “original indication” (OI) and the secondary disease as the “new indication” (NI). DR can reduce costs associated with developing a new drug, and since data would be available for both safety and pharmacokinetics, Phase I trials could be omitted and thus reduce development time.^2^ Given these features, DR is suited for developing treatments during an epidemic. Less funding would be required for DR in an emergency relative to that required to develop a new chemical entity (NCE) with relatively high treatment efficacy. As a valid option in the early stages of an emergency, when research funds are not immediately available, DR can bridge the gap until NCE development.^3^

**Table 1.** Conditions Which Pave the Way for Drug Repositioning Resource Allocation Issues

Irreplaceable	The drug of interest plays a deciding role in determining the health of the OI patient; that is, the drug is a standard treatment needed to improve mortality, and alternative treatments are markedly inferior	
Unobtainable	A barrier exists that physically prevents the OI patient from obtaining the drug of interest	
	Large demand	The amount used by the NI is large, for example, when the drug is used for mild/moderate NI cases and/or for prevention
	Short supply	The supply is markedly low and time is required to replenish it
	Logistical barriers	A lockdown is in place such that domestic and international commodity distribution has come to a standstill
Financial pressure	The target drug is expensive and will present a massive burden on emergency medical finances, putting other emergency strategies at risk	

Abbreviations: NI, new indication; OI, original indication.

Our prior experiences with severe acute respiratory syndrome coronavirus (SARS-CoV) and Middle East respiratory syndrome coronavirus (MERS-CoV)^4^ have taught us that anti-HIV drugs such as lopinavir/ritonavir may be effective against coronaviruses.^5^ China, followed by countries including Thailand and Japan, began using lopinavir/ritonavir as an experimental treatment for COVID-19^6^ and have even conducted randomized controlled trials (RCTs). In addition, remdesivir, which was developed to treat Ebola virus infections, as well as favipiravir, an anti-influenza drug, are also considered potentially effective treatment options.^7^ Of the 30 candidate COVID-19 drugs selected for consideration by the Shanghai Institute of Materia Medica, 12 were anti-HIV drugs.^8^ In this manner, DR has been considered for numerous drugs from the early stages of the COVID-19 outbreak.^9^

One important ethical consideration when repurposing these drugs is the impact it would have on certain social groups. Including the COVID-19 cases from the Diamond Princess cruise ship, Japan had a high number of cases early on and was faced with a demand for COVID-19 treatment, which initiated discussions on DR. As the world eagerly awaits novel treatments for COVID-19, treatments from DR are likely to become globally prevalent. However, no study has addressed the issue of DR in emergency situations from a broader perspective, taking into account the social and ethical ramifications. Against this backdrop, the present paper analyzes social and ethical issues surrounding DR in emergency situations with a focus on Japan, and presents lessons we might learn from these.

## Fairness in Resource Distribution for DR in Emergency Situations: COVID-19 is Not the Only Disease

When considering DR in emergency situations, one issue that requires ethical consideration is the fair distribution of resources (i.e., drugs) among OI and NI patients. Ezekiel Emanuel et al. (when author introduced in text include first name) described the ethical challenge of allocating medical resources (specifically, respirators) for COVID-19.^10^ However, in terms of medical resource distribution for DR in emergency situations, the problem is not allocation among patients with COVID-19, but rather allocation between COVID-19 patients and those with other diseases. Examining the ethics of resource distribution in this situation requires a more macroscopic perspective.

According to a case report from Japan, ciclesonide, an inhaled steroid for asthma as the OI, was found to be effective in treating COVID-19.^11^ The pharmaceutical company in Japan serving as the distribution source was subsequently bombarded by requests from medical institutions and pharmacies. The company expressed its concern for the demand, indicating it wanted to avoid a situation in which the supply required by existing asthma patients became inadequate.^12^ This concern became a reality; in conjunction with the DR of ciclesonide to treat COVID-19, the supply to asthma patients who had been using this medication all along became difficult to maintain in some regions of Japan.^13^ As a result, the Japan Association for Infectious Diseases issued a notice stating that the drug should not be used to prevent COVID-19 infections.

Another shortage case was seen in bacillus Calmette-Guérin (BCG) vaccine. After an online preprint article, which suggested that tuberculosis-preventive BCG vaccine could have efficacy of lowering SARS-CoV-2 infection rate^14^ was circulated, demand for this vaccine soared in Japan. BCG is essential for tuberculosis control in a tuberculosis high- or middle-burden countries including Japan. However, BCG vaccine was introduced in 1951, and those born before the introduction may have not vaccinated with this vaccine. As shortages of BCG vaccines were observed in some clinics,^15^ the Japanese Society for Vaccinology released a statement that BCG vaccines for tuberculosis prevention among children should be the priority.^16^

Consumption of anti-HIV drugs in large quantities, especially in cities and countries in lockdown, can deplete the supply of anti-HIV drugs required by HIV carriers. Of the average market share for antiretroviral drugs for children in 2020 in low- and middle-income countries, the share estimate for the protease inhibitor lopinavir/ritonavir was 99%. Estimating this to correspond to 60% for adults,^17^ it is immediately clear that this would significantly impact HIV carriers.

The same phenomenon holds true for chloroquine, a drug conventionally used to treat and prevent malaria. It is also the first-choice medication for systemic lupus erythematosus (SLE), which is also an OI for the drug. Given reports that chloroquine might be effective against COVID-19,^18^ DR without restriction could lead to a similar situation for malaria and SLE patients.

In this manner, DR in emergency situations could deprive OI patients from their necessary medications or vaccines. This very phenomenon is occurring in Japan.

## Conditions Which Pave the Way for DR Resource Distribution Issues

Under which circumstances are issues likely to emerge with regard to DR-related resource distribution? Several noteworthy circumstances are described (Table 1).

In the above circumstances, the rights of OI patients to receive treatment must be considered. In particular, resource distribution issues emerge when the first two conditions (Irreplaceable and Unobtainable) are met. In particular, a drug becomes Unobtainable when Short supply, Large demand, and Logistical barriers are present.

Favipiravir, an anti-influenza medication, was reported to be potentially effective against COVID-19. In order to prepare for the new flu season, the Japanese government decided to store enough favipiravir to treat 2 million people.^19^ Thus, even when the COVID-19 epidemic hit, favipiravir was available in a more than ample supply as a medical resource. In addition, although favipiravir, which is not typically used in general clinical settings, is approved to treat influenza, it is approved in Japan *only* as treatment against novel or re-emerging influenza for which other primary anti-influenza drugs such as oseltamivir are ineffective.^20^ If DR for a drug such as favipiravir were allowed, the risk of depleting medical resources would be low and thus such drugs would justifiably be DR targets. Unfortunately, *in vitro* studies found favipiravir to be ineffective to control SARS-CoV-2019.^21^ If its clinical efficacy against COVID-19 cannot be demonstrated, and then obviously the drug will lack significance as a DR target.

## A Discussion of Ethics: On the Importance of OI Patient Rights to Receive Treatment

The challenge with resource distribution for DR in an emergency is striking a balance between protecting the rights of OI patients to continue to receive treatment and the social demand for repurposed drugs to suppress the emergency. Why should OI patient rights be considered equally important as using those drugs to address the emergency? There are at least two reasons: involuntary risk burden and differences in degree of the quality of evidence.

### High Level of Evidence for OI and Regional Limitations of Clinical Research Ethics During Emergency Situations

First, a high-level quality of evidence for the OI has already been established. The level of medical evidence supporting the effectiveness of drugs for a particular indication is stronger for the OI than for the NI. This is because well-designed, controlled RCTs have been conducted for the OI, and large-scale retrospective analyses using real world data are available, with guaranteed results on long-term efficacy. On the other hand, only low-quality studies conducted in the early stages of a pandemic are likely to serve as the basis for large-scale use of the drugs for the NI. Prioritizing indications with poor medical evidence in this manner violates the principles of evidence-based medicine and is scientifically unsound.

A stringent ethical review is important for maintaining scientific and ethical validity, but not always feasible, particularly in emergencies. Yet, if the emergency is prioritized above everything else, the research is less likely to be well designed, the testing of drug efficacy becomes scientifically inadequate, and the level of evidence decreases. DR in this context unfortunately becomes a simple off-label use of a drug, which lacks both ethical and scientific backing.

Even if Institutional Review Board (IRB) review were feasible for DR in emergency situations, the regional limitations of the IRB would be problematic. For instance, the decision to approve a study at one facility would be made according to the particular situation at that facility. When applied to a wider geographical range or the entire nation, for example, problems with resource distribution would be inevitable. For instance, facility-dependent variations in IRB decisions could create an unfair situation in which some patients are permitted to receive the asthma medication, whereas others are not. Therefore, for DR in emergency situations, simply entrusting the decisions to the IRB at each facility may not achieve fair resource distribution at the macro level of public health. Rather, DR in emergency situations, ethical decisions for approval of DR as an off-label use, should involve an assessment of total resource quantity.

### Involuntary Risk Burden

Without waiting for evidence and moving forward with large-scale DR with only a loose hypothesis regarding efficacy may appear to be an empiric therapeutic strategy for infectious diseases, but this would burden OI patients with involuntary risk. Consequentialism and utilitarianism, two powerful ethical theories pertaining to resource distribution, both advocate for maximizing the resulting benefit as their basis for evaluation. However, when emergency DR causes such an extreme imbalance between supply and demand for drugs, the rights of OI patients to receive treatment are threatened. Even if the DR is later found to be effective, moving forward with the plan while knowing that OI patients might be deprived of their medication goes against John Stuart Mills’ harm principle that people should be free to act unless their actions harm others.^22^ Nor could it be justified solely from the perspective of consequentialism. According to consequentialist reasoning, having calculated the benefits, if mortality among the population as a whole is reduced by the lower mortality rate among NI patients despite the increased mortality rate among OI patients resulting from terminating their treatment, then OI patients would be asked to consent to give up their treatment. For OI patients to be faced with this dilemma, that is, having to donate their medication that should have been available for their use, is simply unjust. This dilemma is similar to the argument concerning living transplantation of the liver and kidneys. That is, although delegation would clearly reduce mortality of the population as a whole, it is not made a compulsory act since it would potentially increase mortality among donors due to surgical complications. It is difficult, not to mention unacceptable, to force consent that would result in an increased mortality risk for a single individual based solely on the potential to reduce mortality of the whole.

With regard to DR of HIV drugs, HIV-infected individuals, who are both socially^23^ and immunologically vulnerable, face further disadvantages. In their guidance for COVID-19, Public Health England noted that clinicians should watch for atypical symptoms in patients who are immunocompromised and consider admission to the hospital.^24^ However, HIV-infected individuals were excluded from clinical trials in the early phase of the pandemic.^25^ Accordingly, DR of anti-HIV drugs has led to the underrepresentation of this vulnerable population in clinical trials. In this way, exclusion of patients with lower immunity from clinical trials further violates the rights of the vulnerable, and is not ethically permissible.

## Roadmaps

When dealing with DR in emergency settings, we must be careful to prioritize the justice of resource distribution. In other words, we must strive to ensure that medications that are already required by patient populations are not stripped from these patients. COVID-19 is not the only disease that can strongly affect humans. OI patients are a medically vulnerable population. In the example of chloroquine, termination of SLE treatment would lead to acute exacerbation of the disease among lupus patients, leaving them to face an increased risk of lupus nephritis or encephalitis. With lopinavir/ritonavir, termination of HIV treatment would lead to higher levels of HIV disease among carriers. As OI patients are not only medically vulnerable, but also socially vulnerable in emergency situations, their rights must be proactively protected. We must not forget that there are OI patients who require the drugs targeted for DR.

In order to ensure that repurposed drugs can be used for both OIs and NIs, data on resource quantities are critically needed. First, we must understand the scale and number of OI patients who require them. Healthcare claims data could be used to this end. Next, we must determine the amount of repurposed drugs stored in various facilities. This would require the cooperation of pharmacies, as well as inventory surveys of pharmaceutical companies and distributors of medical goods. Finally, we would need to gauge the turnaround time required for a pharmaceutical company to produce additional quantities of a given drug.

Only when these data are available can appropriate resource distribution begin. After securing the amount required by OI patients, if deemed medically appropriate to substitute another drug, then this substitution should be pursued. Next, adhering to appropriate protocols, any extras should be supplied as repurposed drugs for DR clinical trials for the purpose of COVID-19 drug discovery. Since COVID-19 is a new infectious disease, administration of any drug to treat these cases would be “first-in-human.” Therefore, any and all DR must be conducted within a solid clinical research framework, and existing clinical research ethical principles would obviously apply. In this process, it will be important to administer the drugs to severe cases first. Administration for preventive purposes should be done only after careful consideration. By the time efficacy is demonstrated in clinical trials, reinforcements to the existing system should have been made to enable additional production. If effectiveness is confirmed, then the additionally produced repurposed drugs can be administered at treatment frontlines for COVID-19.

When DR is employed in emergency situations, the fairness of resource distribution becomes an issue that requires careful ethical consideration. This issue emerges in situations for which the OI drug is irreplaceable and unobtainable. Ethical issues pertaining to DR in emergencies include maintaining the evidence level and integrity of clinical research ethics, application of Mills’ Harm principle to OI patients, and voluntary consent by OI patients. In order to address these ethical issues, rapid accumulation of ethically and scientifically valid evidence is required, as is obtaining information on resource quantity. In order to achieve this, global collaboration is critical.

## Note

1. WHO. Virtual press conference on COVID-19. March 11, 2020; available at https://www.who.int/docs/default-source/coronaviruse/transcripts/who-audio-emergencies-coronavirus-press-conference-full-and-final-11mar2020.pdf (last accessed 14 March 2020).

2.

Chong CR , Sullivan DJ . New uses for old drugs. Nature 2007;448(7154):645–61768730310.1038/448645a. See also, Nosengo N. Can you teach old drugs new tricks? Nature 2016;534(7607):314–62730617110.1038/534314a. See also, Ashburn TT , Thor KB . Drug repositioning: Identifying and developing new uses for existing drugs. Nature Reviews Drug Discovery 2004;3(8):673–83.1528673410.1038/nrd1468

3. WHO. *An R&D Blueprint for Action to Prevent Epidemics: Funding & Coordination Models for Preparedness and Response*; 2016 May; available at https://www.who.int/blueprint/what/improving-coordination/workstream_5_document_on_financing.pdf (last accessed 14 March 2020).

4.

Chu CM . Role of lopinavir/ritonavir in the treatment of SARS: Initial virological and clinical findings. Thorax 2004;59:252–56.1498556510.1136/thorax.2003.012658PMC1746980

5.

Arabi YM , Asiri AY , Assiri AM , Aziz Jokhdar HA , Alothman A , Balkhy HH , et al. Treatment of Middle East respiratory syndrome with a combination of lopinavir-ritonavir and interferon-β1b (MIRACLE trial): Study protocol for a randomized controlled trial. Trials 2018;19:81 2938239110.1186/s13063-017-2427-0PMC5791210. See also, Sheahan TP , Sims AC , Leist SR , Schafer A , Won J , Brown AJ , et al. Comparative therapeutic efficacy of remdesivir and combination lopinavir, ritonavir, and interferon beta against MERS-CoV. Nature Communications 2020;11:222.10.1038/s41467-019-13940-6PMC695430231924756

6.

Huang C , Wang Y , Li X , Ren L , Zhao J , Hu Y , et al. Clinical features of patients infected with 2019 novel coronavirus in Wuhan, China. The Lancet 2020;395(10223):497–506 10.1016/S0140-6736(20)30183-5PMC715929931986264. See also, Nikkei. *Shin-gata haien, kou-HIV-yaku touyo-go ni syojo kaizen [2019-nCoV pneumonia has improved after anti-HIV drugs was given].* 2020 February 6; available at https://www.nikkei.com/article/DGXMZO55341640W0A200C2CC1000/ (last accessed 14 March 2020). See also, Reuters. *Cocktail of Flu, HIV Drugs Appears to Help Fight Coronavirus: Thai Doctors*; 2020 February 2; available at https://www.reuters.com/article/us-china-health-thailand/cocktail-of-flu-hiv-drugs-appears-to-help-fight-coronavirus-thai-doctors-idUSKBN1ZW0GQ (last accessed 14 March 2020).

7. Li G, De Clercq E. Therapeutic options for the 2019 novel coronavirus (2019-nCoV). *Nature Reviews Drug Discovery*; 2020 February 10; available at https://www.nature.com/articles/d41573-020-00016-0 (last accessed 14 March 2020).

8. Shanghai Institute of Materia Medica, Chinese Academy of Sciences. *A Group of Old and Traditional Chinese Medicines that May have a Therapeutic Effect on New Pneumonia*; 2020 January 25; available at http://www.simm.ac.cn/xwzx/kydt/202001/t20200125_5494417.html (last accessed 29 Jan 2020).

9. Harrison C. Coronavirus puts drug repurposing on the fast track. *Nature Biotechnology*; 2020 February 27; available at https://www.nature.com/articles/d41587-020-00003-1 (last accessed 14 March 2020). See also, Fletcher ER. Over 30 antiviral drugs being tested against novel coronavirus: As WHO Convenes Global Innovation Forum. *Health Policy Watch*; 2020 February 10; available at https://www.healthpolicy-watch.org/over-30-antiviral-drugs-being-tested-against-against-novel-coronavirus-as-who-convenes-global-innovation-forum/ (last accessed 11 March 2020).

10. Emanuel EJ, Persad G, Upshur R, Thome B, Parker M, Glickman A, et al. Fair allocation of scarce medical resources in the time of Covid-19. *The New England Journal of Medicine*; 2020 March 23; available at https://doi.org/10.1056/NEJMsb2005114 (last accessed 14 March 2020).

11. Iwabuchi K, Yoshie K, Kuraue Y, Takahashi Y, Kato Y, Morishima T. et al. *COVID-19 haien syoki kara chuki ni sikuresonido kyunyu wo siyou si kaizen sita 3 rei [Three Cases of Recovery from Early to Middle COVID-19 Pneumonia after Inhaling Ciclesonide]*; 2020 March 2; available at http://www.kansensho.or.jp/uploads/files/topics/2019ncov/covid19_casereport_200302_02.pdf (last accessed 12 March, 2020).

12. m3. *COVID-19 heno sikuresonido, ippan rinsyo de siyou muzukasii 2 tu no riyu. [2 Hard Issues Prevent Using Ciclesonide for COVID-19 in Clinical Settings] m3*; 2020 March 6; available at https://www.m3.com/clinical/news/735718 (last accessed 12 March 2020).

13. The Japan Association for Infectious Diseases. *Sikuresonido siyo-jo no go-chui [Precautions for the Usage of Ciclesonide]*; 2020 March 16; available at http://www.kansensho.or.jp/uploads/files/topics/2019ncov/covid19_note_ciclesonide.pdf (last accessed 12 March 2020).

14. De Vrieze J. Can a century-old TB vaccine steel the immune system against the new coronavirus? *Science*; 2020 March 23; available at https://www.sciencemag.org/news/2020/03/can-century-old-tb-vaccine-steel-immune-system-against-new-coronavirus (last accessed 12 March 2020).

15. Sakamaoto N. BCG Korona ni yuuko? Kasetsu de otona no sesshu-kibo kyuzo. Nyuji he kyoukyu ni eikyo mo [Is BCG vaccine effective for COVID-19? A hypothesis made adults request for vaccination. Impact on supply for infants would be seen]. *Nishinippon Shimbun*; 2020 April 8; available at https://www.nishinippon.co.jp/item/n/599010/ (last accessed 12 March 2020).

16. The Japanese Society for Vaccinology. *Sin-gata korona-uirusu kansensho ni tai-suru BCG wakuchin no kouka ni kan-suru kenkai [An Opinion for the Effectiveness of BCG Vaccine against COVID-19]*; 2020 April 3; available at http://www.jsvac.jp/pdfs/kenkai.pdf (last accessed 4 April 2020).

17. World Health Organization. *Combined Global Demand Forecasts for Antiretroviral Medicines and HIV Diagnostics in Low- and Middle-Income Countries from 2015 to 2020*; 2016 September; available at https://apps.who.int/iris/bitstream/handle/10665/250088/9789241511322-eng.pdf (last accessed 12 March 2020).

18.

Wang M , Cao R , Zhang L , Yang X , Liu J , Xu M , et al. Remdesivir and chloroquine effectively inhibit the recently emerged novel coronavirus (2019-nCoV) in vitro. Cell Research 2020;30:269–71.3202002910.1038/s41422-020-0282-0PMC7054408

19. Shin-gata inhuruenza-tou oyobi tori-inhuruenza-tou ni kan-suru kankei syocho taisaku kaigi. Shin-gata inhuruenza-tou taisaku gaidorain [Guideline of countermeasures for new types of influenza and other emerging diseases]. *Cabinet Secretariat*; 2013 June 26; available at https://www.cas.go.jp/jp/seisaku/ful/keikaku/pdf/h300621gl_guideline.pdf (last accessed 14 February 2020).

20. FUJIFILM Toyama Chemical Co., Ltd. *Avigan-jyo 200 mg*; 2019 April; available at http://fftc.fujifilm.co.jp/med/abigan/pack/pdf/abigan_package_01.pdf (last accessed 14 February 2020).

21. See note 18, Wang et al. 2020.

22.

Mill JS . On liberty In: Robson JM and Brady A , eds. Essays on Politics and Society. Toronto, Buffalo: University of Toronto Press; 1977, at 213–310.

23.

Zhang L , Chow EPF , Jing J , Zhuang X , Li X , He M , et al. HIV prevalence in China: Integration of surveillance data and a systematic review. The Lancet Infectious Disease 2013;13:955–63.10.1016/S1473-3099(13)70245-724107261

24. Public Health England. *Guidance: COVID-19: Investigation and Initial Clinical Management of Possible Cases*; available at https://www.gov.uk/government/publications/wuhan-novel-coronavirus-initial-investigation-of-possible-cases/investigation-and-initial-clinical-management-of-possible-cases-of-wuhan-novel-coronavirus-wn-cov-infection (last accessed 12 March 2020).

25. Chinese Clinical Trial Registry. *A Randomized, Open-Label, Blank-Controlled Trial for the Efficacy and Safety of Lopinavir-Ritonavir and Interferon-Alpha 2b in Hospitalization Patients with Novel Coronavirus Infection.* Chengdu, China: Ministry of Health (China), 2020. Identifier ChiCTR2000029308; available at http://www.chictr.org.cn/showproj.aspx?proj=48684 (last accessed 29 Jan 2020).

